# HMGA1 stimulates MYH9-dependent ubiquitination of GSK-3β via PI3K/Akt/c-Jun signaling to promote malignant progression and chemoresistance in gliomas

**DOI:** 10.1038/s41419-021-04440-x

**Published:** 2021-12-10

**Authors:** Tianshi Que, Haojie Zheng, Yu Zeng, Xinru Liu, Ge Qi, Qingcuo La, Tuo Liang, Zhiyong Li, Guozhong Yi, Shichao Zhang, Junjie Li, Jing Nie, Jian-er Tan, Guanglong Huang

**Affiliations:** 1 Department of Neurosurgery, Nanfang Hospital, Southern Medical University, Guangzhou, 510515 Guangdong People’s Republic of China; 2 The Laboratory for Precision Neurosurgery, Nanfang Hospital, Southern Medical University, Guangzhou, 510515 Guangdong People’s Republic of China; 3 Nanfang Glioma Center, Nanfang Hospital, Southern Medical University, Guangzhou, 510515 Guangdong People’s Republic of China; 4 Department of Cell Biology, School of Basic Medical Sciences, Southern Medical University, Guangzhou, 510515 Guangdong People’s Republic of China; 5 The First Clinical Medical School, Southern Medical University, Guangzhou, 510515 Guangdong People’s Republic of China; 6 School of Pharmaceutical Sciences, Southern Medical University, Guangzhou, 510515 Guangdong People’s Republic of China; 7 Nanfang PET Center, Nanfang Hospital, Southern Medical University, Guangzhou, 510515 Guangdong People’s Republic of China

**Keywords:** CNS cancer, Cell signalling

## Abstract

Myosin heavy chain 9 (MYH9) plays an essential role in human diseases, including multiple cancers; however, little is known about its role in gliomas. In the present study, we revealed that HMGA1 and MYH9 were upregulated in gliomas and their expression correlated with WHO grade, and HMGA1 promoted the acquisition of malignant phenotypes and chemoresistance of glioma cells by regulating the expression of MYH9 through c-Jun-mediated transcription. Moreover, MYH9 interacted with GSK-3β to inhibit the expression of GSK-3β protein by promoting its ubiquitination; the downregulation of GSK-3β subsequently promoted the nuclear translocation of β-catenin, enhancing growth, invasion, migration, and temozolomide resistance in glioma cells. Expression levels of HMGA1 and MYH9 were significantly correlated with patient survival and should be considered as independent prognostic factors. Our findings provide new insights into the role of HMGA1 and MYH9 in gliomagenesis and suggest the potential application of HMGA1 and MYH9 in cancer therapy in the future.

## Introduction

Glioma is the most common primary brain malignancy. The current main therapy is maximal safe resection, followed by radiotherapy in combination with chemotherapy [1–4]. Despite consistent progress in treatment, the therapeutic effect is barely ameliorated. Owing to its high malignancy, glioma is hard to cure and easily recurrent. High malignancy indicates rapid and infiltrative growth, formidable invasive and migrant capability of tumor cells. Moreover, the malignant progression of gliomas is hypothesized to be induced by chemotherapy resistance. Therefore, it is an important direction to study high proliferation, invasion, and migration, as well as chemoresistance in gliomas.

A collection of studies recently demonstrated that high mobility group AT-hook 1 (HMGA1), which acts as a tumor promoter, is associated with several types of human cancers, including non-small cell lung cancer [5], breast cancer [6], and cervical cancer [7]. Recent studies have suggested the important roles of HMGA1 in gliomas. Its expression correlated significantly with glioma malignancy, proliferation, invasion, and angiogenesis of gliomas [8, 9]. Although we have previously identified an oncogenic ZEB2/miR-637/HMGA1 signaling axis targeting Vimentin which could promote both migration and invasion in glioma [10], little is known about the role of HMGA1 which is worthy of continuous investigation.

Myosin heavy chain 9 (MYH9), which belongs to Myosin family, is involved in cell adhesion and migration [11] and plays essential roles in multiple human diseases [12]. It is overexpressed in multiple cancers, including gastric cancer [13], non-small cell lung cancer [14], colon carcinoma [15], and breast cancer [16]. It promotes the tumorigenesis and development of those cancers and has a positive correlation with the prognosis of patients. Recently, it was confirmed that MYH9 binds to the CTNNB1 promoter to promote CTNNB1 transcription, conferring resistance to anoikis in gastric cancer [17]. Targeting MYH9 blocked HBX-induced GSK-3β ubiquitination to activate the β-catenin destruction complex, suppressing hepatocellular cancer stemness and the epithelial-to-mesenchymal transition [18]. For gliomas, overexpression of MYH9 contributes to cell migration ability [19], suggesting major roles in cell migration and tumor invasion [20]. However, the expression and role of MYH9 in gliomas are still undetermined.

In the present study, we showed that upregulation of HMGA1 and MYH9 in human gliomas is correlated with tumor progression, and knockdown of HMGA1 can inhibit the proliferation, migration, and invasion, as well as chemoresistance in glioma cells by stimulating MYH9-mediated ubiquitinated degradation of GSK-3β through PI3K/Akt/c-Jun pathway in vitro and in vivo. Our data tend to provide new insights into the molecular function of HMGA1 and MYH9 as well as its regulatory mechanisms in gliomas.

## Results

### Upregulation of HMGA1 and MYH9 is correlated with glioma progression

**Fig. 1:**
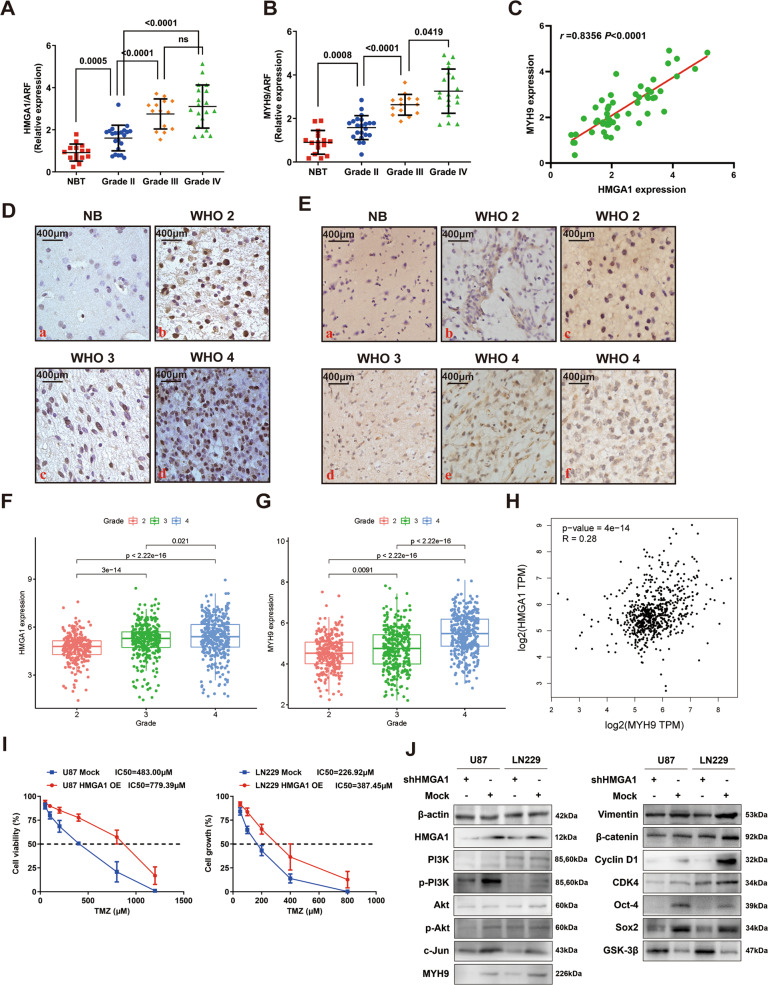
Upregulation of HMGA1 and MYH9 was correlated with the progression of gliomas. **A**, **B** HMGA1 (**A**) and MYH9 (**B**) mRNA expression were upregulated in glioma tissues compared to normal brain tissues, and overexpressed levels of HMGA1 and MYH9 in glioma patients were positively correlated with the status of pathological classification. **C** The expression of HMGA1 was positively related to MYH9 expression. **D** Immunohistochemistry assay identified HMGA1 was overexpressed in glioma sample tissues compared to normal brain tissues. **E** MYH9 was overexpressed in glioma sample tissues. Strong expression of MYH9 was mainly observed in cytoplasmic (**B**, **D**, and **E**) and partly found in the nucleus (**C** and **F**) in glioma tissues. **F**, **G** Analysis of Chinese Glioma Genome Atlas (CCGA) database suggested HMGA1 (**F**) and MYH9 (**G**) mRNA level was significantly increased with the increase of WHO grade. **H** The expression of HMGA1 was positively related to MYH9 expression in CCGA database. **I** Dose–response curves of TMZ treatment were examined for HMGA1-overexpression groups and the control groups. **J** Western blot assay confirmed the change of the downstream pathway of HMGA1.

To determine the roles of HMGA1 and MYH9 in gliomagenesis, the protein and mRNA expression of HMGA1 and MYH9 in clinical sample tissues were detected by quantitative real-time PCR (qPCR) and immunohistochemistry (IHC) assays. QPCR showed that both HMGA1 and MYH9 mRNA levels were upregulated in glioma tissues compared to normal brain tissues (NB), and overexpressed levels of HMGA1 and MYH9 in glioma patients were positively correlated with pathological classification (WHO 2 vs. WHO 3 vs. WHO 4) (*P* < 0.05) (Fig. 1A and B). Further analysis confirmed HMGA1 expression was positive correlated with MYH9 expression (*r* = 0.8356, *P* < 0.0001) (Fig. 1C). IHC revealed that the protein expression of HMGA1 and MYH9 also was increased in glioma tissues compared with NB.

In diffuse astrocytoma and glioblastoma, strong cytoplasmic and nuclei staining for HMGA1 protein was observed, whereas only slight nuclear HMGA1 expression was detected in NB (Fig. 1D). Strong MYH9 staining was primarily observed in the cytoplasm and secondarily in the nucleus in glioma tissues, whereas low expression was observed in NB tissues (Fig. 1E). Subsequently, the data for HMGA1 and MYH9 mRNA expression in gliomas were extracted from the Chinese Glioma Genome Atlas (CCGA) database [21, 22]. Both HMGA1 and MYH9 mRNA levels were significantly increased with increasing WHO grade (Fig. 1F and G), and HMGA1 expression was positively correlated with MYH9 expression (*r* = 0.28, *P* < 0.01) (Fig. 1H). These results suggested that HMGA1 and MYH9 were upregulated in glioma tissues and correlated with the progression of gliomas.

### HMGA1 reduces glioma sensitivity to temozolomide

In our previous work, we demonstrated that overexpressed HMGA1 promoted proliferation, invasion, and migration in glioma cells, and Vimentin could directly bind to HMGA1 [10]. To further determine whether HMGA1 inhibition sensitizes U87 and LN229 cells to TMZ-induced cell death, dose–response TMZ curves were assessed for cells overexpressing HMGA1 and control (Mock) groups (Fig. 1I). The TMZ concentration causing IC_50_ in U87 cells increased from 483.00 to 779.39 μM with HMGA1 overexpression (Fig. 1I, left), while HMGA1 overexpression in LN229 cells increased the TMZ IC_50_ from 226.92 to 387.45 μM (Fig. 1I right). Importantly, we found that knockdown of HMGA1 increased the expression of γ-H2AX, a biomarker for DNA double-strand breaks (Supplemental Fig. 1A). These data suggested that HMGA1 inhibition sensitizes glioblastoma cells to TMZ-induced apoptosis.

Then, a western blot assay was performed to explore the mechanism of HMGA1 (Fig. 1J and Supplemental Fig. 1B). Phosphorylation of PI3K and Akt was inactivated by HMGA1 knockdown. Silencing of HMGA1 downregulated the expression of c-Jun, which is a downstream factor in the PI3K/Akt pathway while upregulating GSK-3β. Moreover, expression of epithelial-mesenchymal transition (EMT)-related factors, including β-catenin and Vimentin, and cell cycle-related proteins such as Cyclin D1 and CDK4, were reduced after inhibition of HMGA1, as well as stemness-related factors Oct-4 and Sox2. Interestingly, we found that the expression of MYH9 was decreased by HMGA1 silencing, indicating that HMGA1 might directly or indirectly regulate MYH9 expression.

### HMGA1 increases MYH9 expression through c-Jun-mediated transcription

**Fig. 2:**
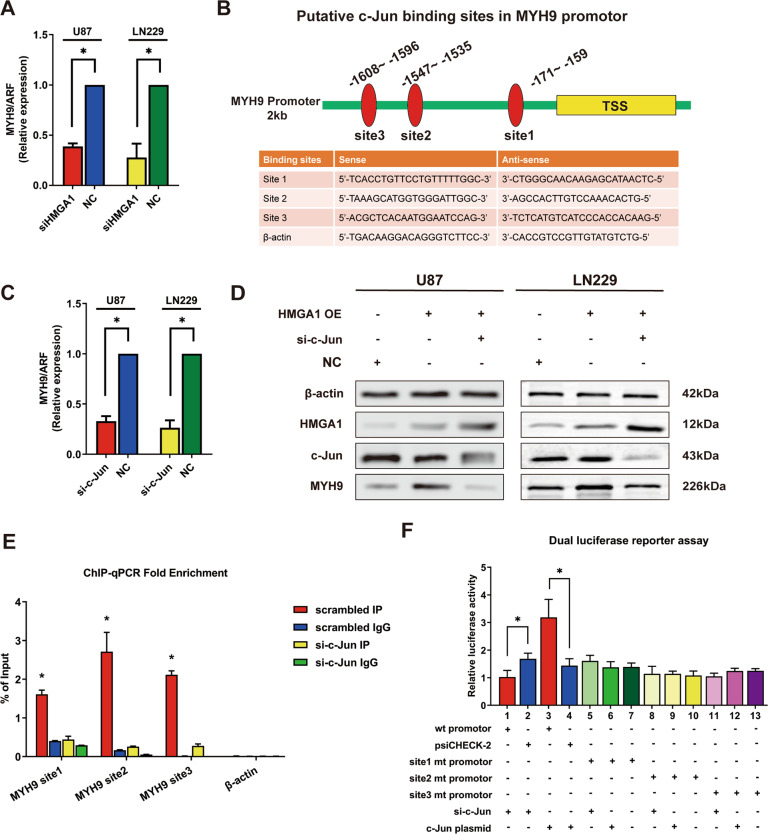
HMGA1 increased the expression of MYH9 through c-Jun-mediated transcription. **A** Knockdown HMGA1 leads to the decreasing of MYH9 mRNA expression. **B** Schematic diagram of putative c-Jun binding sites in MYH9 promotor and the primers of each binding site. **C**, **D** Knockdown c-Jun induced inhibition of MYH9 mRNA (**C**) and protein (**D**) expression. **E** c-Jun bound to all the predicted sites inside the transcription regulatory region of MYH9, and knockdown of c-Jun could inhibit the protein–DNA interactions in all three binding sites. **F** Dual-luciferase reporter assay showed that the reporter plasmid with the promotor region of MYH9 resulted in a significant decrease in luciferase activity after transfection with si-c-Jun, whereas the plasmid without MYH9 promotor had no change in luciferase activity.

To confirm the relationship between HMGA1 and MYH9, we performed qPCR and western blotting. As shown in Fig. 1J and Fig. 2A, HMGA1 silencing led to a decrease in both mRNA and protein expressions of MYH9. However, bioinformatics data suggested that HMGA1, as a transcription factor, could not bind to the promoter region of MYH9 (data was not shown). Using the UCSC Genome Browser and PROMO, we found that the transcription factor c-Jun might bind to the promoter of MYH9, with three c-Jun-binding motifs at −171 to −159 (Site 1), −1547 to −1535 (Site 2), and −1608 to −1596 (Site 3) of the transcription start site present in the promoter region (Fig. 2B).

To validate these findings, we silenced c-Jun expression in glioma cells; and qPCR and western blotting revealed that knockdown of c-Jun markedly reduced MYH9 expression (Fig. 2C and D). Then, ChIP assay was performed to examine potential protein–DNA interactions between c-Jun and the transcription regulatory region of MYH9. We found that c-Jun bound to all predicted sites inside the transcription regulatory region of MYH9 in U87 cells; moreover, downregulation of c-Jun inhibited the protein–DNA interaction at all three binding sites (Fig. 2E and Supplemental Fig. 1C). A dual-luciferase reporter assay was performed to confirm whether c-Jun positively mediates the expression of MYH9. We subcloned the promoter region of MYH9, including the predicted c-Jun recognition site or mutated sequences into luciferase reporter plasmids. The reporter plasmid with the promoter region of MYH9 resulted in a significant decrease in luciferase activity after transfection with si-c-Jun, whereas the plasmid without the MYH9 promoter showed no change in luciferase activity (Fig. 2F).

### MYH9 promotes proliferation, invasion, migration, and TMZ resistance in gliomas

**Fig. 3:**
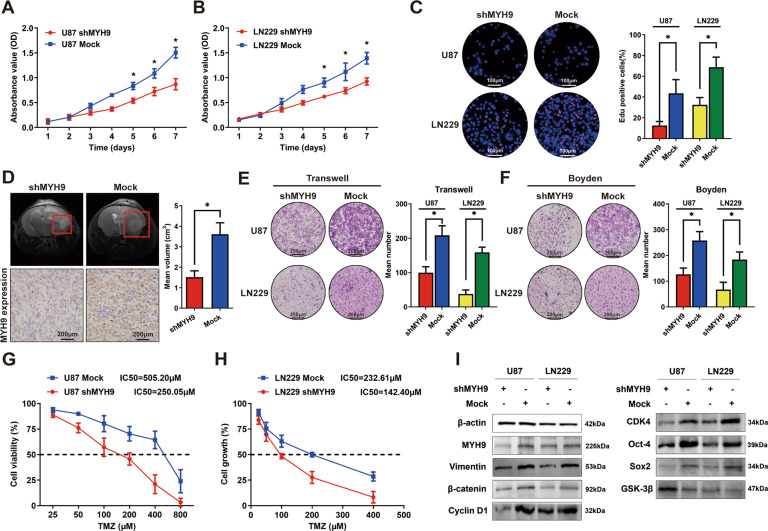
MYH9 promoted proliferation, invasion, migration, and TMZ resistance in gliomas. **A**, **B** MTT assays showed MYH9 downregulation observably inhibited the cell growth. **C** Edu incorporation assays showed knockdown of MYH9 decreased the cell proliferation by blocking G1/S phase transition in glioma cells. **D** Intracranial orthotopic transplantation suggested knockdown of MYH9 inhibited the tumorigenesis in gliomas. **E**, **F** Transwell (**E**) and Boyden (**F**) chamber assays confirmed knockdown of MYH9 inhibited the ability of cell invasion and migration. **G**, **H** Dose–response curves were delineated for TMZ treatment in shMYH9 cells and the control groups to identify the role of MYH9 in chemoresistance. **I** Western blot assay confirmed the change of the downstream pathway of MYH9.

We transfected U87 and LN229 cells with a lentiviral vector carrying the MYH9-shRNA vector (shMYH9) and a control vector (Mock). 3-(4,5-Dimethylthiazol-2-yl)−2,5-diphenyltetrazolium bromide (MTT) assays (Fig. 3A and B) and Edu incorporation assays (Fig. 3C) showed that MYH9 downregulation inhibited cell growth by blocking the G1/S phase transition. U87-shMYH9 cells and control cells were injected into the cerebrum of nude mice. The mice injected with U87-shMYH9 cells had a smaller mean tumor volume than controls (Fig. 3D). Transwell and Boyden chamber assays revealed that MYH9 significantly increased invasiveness and migration over controls (Fig. 3E and F). TMZ dose–response curves showed that the IC_50_ in U87 cells decreased from 505.20 to 250.05 μM after MYH9 knockdown (Fig. 3G); MYH9 knockdown in LN229 cells reduced the IC_50_ from 232.61 to 142.40 μM (Fig. 3H). We used the MYH9-overexpression vector (MYH9 OE) to identify the phenotype of U87 and LN229 cells after functional recovery of MYH9, and the results also suggested that MYH9 promoted proliferation, invasion, migration, and chemotherapy resistance in glioma cells (Supplemental Fig. 2).

Subsequently, western blotting was performed to explore the mechanism of MYH9 and to find the downstream pathway. Inhibition of MYH9 led to the upregulation of GSK-3β expression in both U87 and LN229 cells, similar to the effect of HMGA1 (Fig. 3I). The expression of EMT-related proteins such as β-catenin and vimentin, cell cycle-related factors including Cyclin D1 and CDK4, as well as stemness-related factors including Oct-4 and Sox2 were all downregulated by silencing MYH9. Moreover, the MYH9 knockdown increased the expression of γ-H2AX, suggesting the enhancement of double-strand DNA breaks (Supplemental Fig. 1D).

### HMGA1 promotes malignant phenotypes and chemoresistance of glioma cells via MYH9

**Fig. 4:**
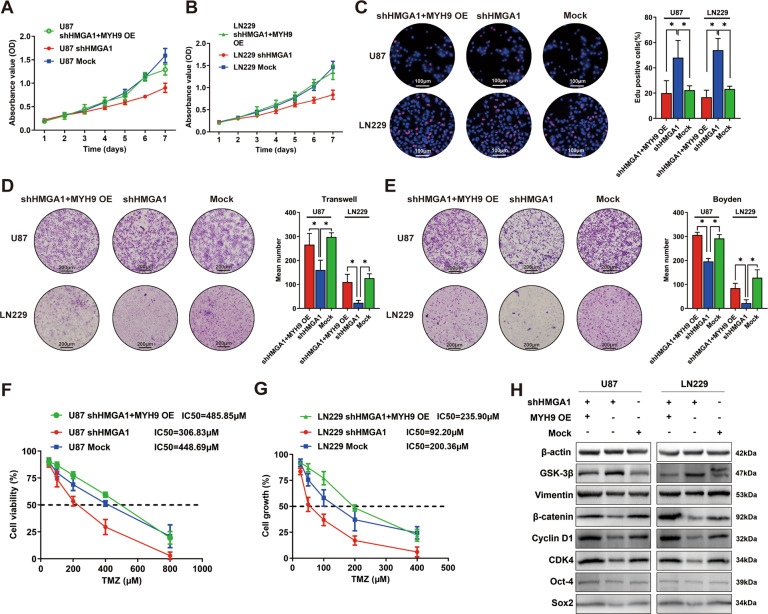
HMGA1 promoted malignant phenotypes and chemoresistance of glioma cells via MYH9. **A**–**C** MTT (**A** and **B**) and Edu incorporation (**C**) assays showed that MYH9 upregulation reversed the shHMGA1-mediated reduction of growth ability in glioma cells. **D**, **E** Transwell (**D**) and Boyden (**E**) chamber assays verified that the ability of cell invasion and migration was recovered in shHMGA1+ MYH9 plasmid-treated cells group compared to shHMGA1-treated cells group. **F**, **G** Dose–response curves for TMZ showed the TMZ concentration causing IC_50_ was rescued in shHMGA1+MYH9 plasmid-treated cells groups compared to shHMGA1-treated cells groups. **H** Western blot assay confirmed MYH9 upregulation rescued shHMGA1-mediated reduction of the EMT-related proteins, the cell cycle-related factors, and the stemness-related factors, and MYH9-overexpression could reverse the shHMGA1-mediated increase of GSK-3β.

To confirm whether HMGA1 modulating of MYH9 was responsible for the promotion of the proliferation, migration, and invasion of glioma cells, we utilized an MYH9-overexpression vector in shHMGA1 glioma cells. MTT and Edu incorporation assays showed that MYH9 upregulation reversed the shHMGA1-mediated reduction of proliferation in glioma cells (Fig. 4A–C). Transwell chamber invasion and Boyden chamber migration assays verified that the invasive and migratory abilities were increased in the shHMGA1 + MYH9 plasmid-treated cells group compared to the shHMGA1-treated cells group (Fig. 4D and E). To determine whether MYH9 reversed the shHMGA1-mediated reduction of TMZ-induced cell death, dose–response curves for TMZ showed the IC_50_ was rescued in shHMGA1 + MYH9 plasmid-treated cells relative to shHMGA1-treated cells (Fig. 4F and G).

Subsequently, we examined the expression of several proteins related to the cell cycle, EMT, stemness, and DNA double-strand breaks in shHMGA1-treated cells overexpressing MYH9, compared with shHMGA1-treated and mock cells separately. As shown in Fig. 4H and Supplemental Fig. 1E, MYH9 overexpression reversed the shHMGA1-mediated increase of GSK-3β and rescued the shHMGA1-mediated reduction of EMT-related proteins (β-catenin and vimentin), cell cycle-related factors (cyclin D1 and CDK4), stemness-related factors (Oct-4 and Sox2), and the biomarker of DNA double-strand breaks (γ-H2AX).

### MYH9 interacts with GSK-3β and promotes its ubiquitination

**Fig. 5:**
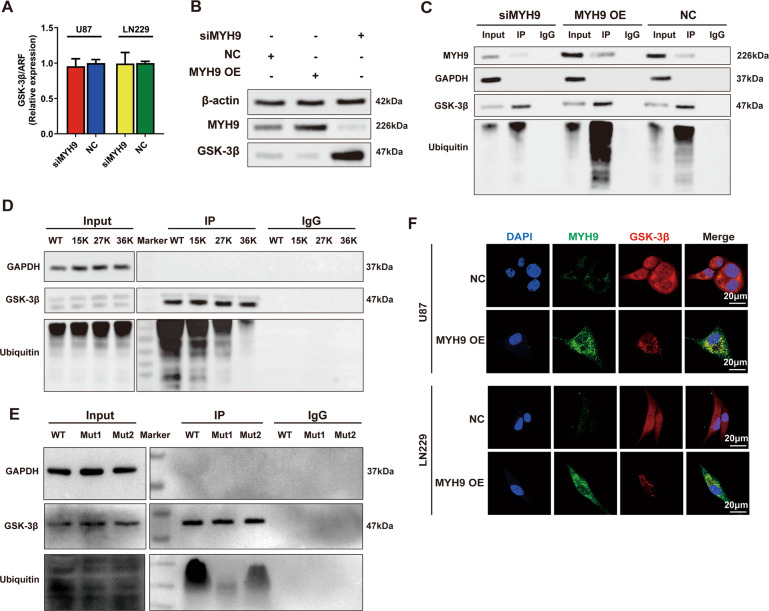
MYH9 interacted with GSK-3β and promotes its ubiquitination. **A** Knockdown of MYH9 had no effect on the GSK-3β mRNA level in glioma cells. **B** Overexpression of MYH9 could downregulate GSK-3β protein level and vice versa. **C** GSK-3β and MYH9 proteins bind to each other, and overexpression of MYH9 downregulated GSK-3β protein level and remarkedly increased Ubiquitin activity. **D** Mutation of all three potential ubiquitination sites could induce the reduction of ubiquitination of GSK-3β. **E** Mutation of two potential interaction domains of MYH9 could both upregulate the GSK-3β protein level and inhibit Ubiquitin activity. **F** Immunofluorescence staining showed MYH9 and GSK-3β both mainly located in the cytoplasm, and overexpression of MYH9 could reduce the cytoplasmic expression of GSK-3β.

Previously, we showed that MYH9 expression affected the protein expression levels of GSK-3β, which could explain the effect of HMGA1 on GSK-3β expression. Then, we sought to identify the relationship between MYH9 and GSK-3β. We first measured the effect of MYH9 on GSK-3β mRNA levels, finding that silencing MYH9 had no effect on GSK-3β mRNA levels in glioma cells (Fig. 5A). However, overexpression of MYH9 downregulated GSK-3β protein levels and vice versa (Fig. 5B), indicating that GSK-3β protein expression was regulated at the post-transcriptional level. DOMINE data sets (http://domine.utdallas.edu/) [23] predicted that MYH9 might interact with GSK-3β, so a co-IP assay was performed. As expected, GSK-3β and MYH9 proteins coprecipitated (Fig. 5C). Overexpression of MYH9 not only downregulated GSK-3β protein levels but also markedly increased ubiquitin activity, suggesting that MYH9 mediates GSK-3β protein levels via ubiquitination. To test this hypothesis, we constructed three different plasmids, each with a mutation changing the lysine residue at three potential ubiquitination sites (15 K, 27 K, and 36 K), then the plasmids with the GSK-3β ubiquitination sites mutant (mt) and wild-type (wt) were transfected into U87 cells. Co-IP showed that all three mutants reduced the ubiquitination of GSK-3β, but the 27 K and 36 K mutant plasmids led to more significant inhibition of ubiquitination, indicating that they are likely the in vivo ubiquitination sites in GSK-3β (Fig. 5D).

We constructed two plasmids with mutated potential interaction domains of MYH9 (Mut1: 75–777, Mut2: 841–1921). Exogenous co-IP indicated that both mutants upregulated GSK-3β protein expression and inhibited ubiquitin activity, but mutation of domain 1 (75–777) displayed a greater suppression of ubiquitin activity (Fig. 5E). Finally, we detected the colocalization of MYH9 and GSK-3β by immunofluorescence staining. MYH9 and GSK-3β were mainly located in the cytoplasm, and overexpression of MYH9 reduced the cytoplasmic expression of GSK-3β (Fig. 5F).

It has been reported that inhibition of GSK-3β promotes the nuclear translocation of non-phosphorylated β-catenin [24]. We also determined whether the ubiquitinated degradation of GSK-3β protein induced by HMGA1/MYH9 signaling promoted the translocation of β-catenin into the nucleus. Immunofluorescent detection of MYH9 and β-catenin was performed on glioma cells overexpressing HMGA1; we observed increased cytoplasmic expression of MYH9 and total expression of β-catenin. Furthermore, we found increased nuclear β-catenin (Supplemental Fig. 3).

### Elevated HMGA1 and MYH9 expression as an unfavorable factor in gliomas

**Fig. 6:**
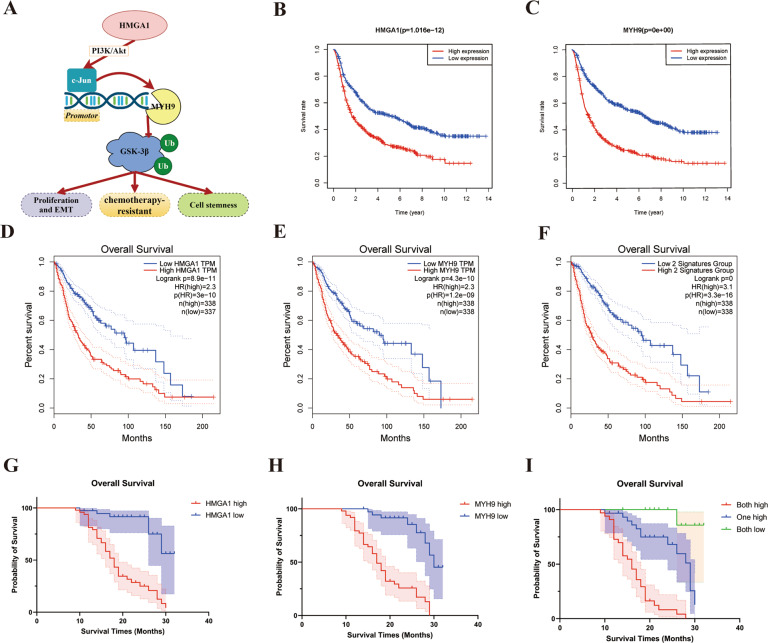
Elevated HMGA1 and MYH9 expression as an unfavorable factor in glioblastoma. **A** A brief illustration of the oncogenic HMGA1/MYH9 signaling pathway. **B**, **C** Survival analysis from CCGA database suggested that mRNA expression level of HMGA1 (**B**) and MYH9 (**C**) had a positive relationship with glioma patients’ survival time. **D**, **E** The results of TCGA database also suggested that patients with low expression of HMGA1 or low expression MYH9 had a longer survival time compared to patients with high expression. **F** Survival analysis from TCGA database showed patients with both high expressions of HMGA1 and MYH9 had significantly worse survival times compared to patients with both low expressions. **G**, **H** Survival analysis about primary glioma cases in our department showed HMGA1 expression and MYH9 expression were found to be positively associated with the overall survival. **I**. Patients with both high expression of HMGA1 and MYH9 had the shortest survival time, whereas patients with both low expressions of HMGA1 and MYH9 had the best survival time.

The oncogenic HMGA1/MYH9 signaling pathway, shown in Fig. 6A, was assessed using the CCGA and The Cancer Genome Atlas (TCGA) databases, as well as using a cohort of 86 prospectively collected primary glioma tissues from our department. Survival analysis based on CCGA and TCGA data suggested that patients with low expression of HMGA1 or low expression MYH9 had a longer survival time compared to patients with high expression (Fig. 6B–E). Moreover, patients with both low expression of HMGA1 and MYH9 had remarkably longer survival times compared to patients with both high expression levels (Fig. 6F). In primary glioma tissues from our department, both HMGA1 and MYH9 were upregulated in glioma samples compared to NB tissues (Table 1). We found that age, histologic type, and WHO grade were significantly correlated with both HMGA1 and MYH9 expression (Table 1).

**Table 1:** Correlation between the clinicopathologic factors and expression of HMGA1 and MYH9 in glioma.

Factors	*n*	HMGA1 expression (%)			MYH9 expression (%)		
		High	Low	*P*	High	Low	*P*
*Tumors and normal samples*							
Tumors	86	48	38	**0.01**	49	37	**0.008**
Normal brain tissues	15	3	12		4	11	
*Gender*							
Male	57	35	22	0.143	36	21	0.105
Female	29	13	16		13	16	
*Age*							
≥50	36	25	11	**0.031**	26	10	**0.015**
<50	50	23	27		23	27	
*Histologic type*							
DA	28	9	19	**<0.001**	7	21	**<0.001**
OD	11	3	8		4	7	
AA	14	10	4		10	4	
GBM	33	26	7		28	5	
*WHO grade*							
II	39	12	27	**<0.001**	11	28	**<0.001**
III	14	10	4		10	4	
IV	33	26	7		28	5	

*DA* diffuse astrocytoma, *OD* oligodendroglioma*,* *AA* anaplastic astrocytoma, *GBM* glioblastoma.

Bold values indicates statistically significant *P* < 0.05 values.

Using Kaplan–Meier analysis and the log-rank test (Table 2), HMGA1 expression, MYH9 expression, age, pathological diagnosis, and WHO grade were found to be significantly associated with the overall survival of glioma patients in univariate analysis (both *P* < 0.05). The mean survival time was only 19.090 ± 0.952 months (95% confidence interval (CI): 17.224–20.955) in the high HMGA1 group and 18.640 ± 0.944 months (95% CI: 16.790–20.490) in the high MYH9 group, whereas they were 28.915 ± 1.069 months (95% CI: 26.819–31.011) in the low HMGA1 group and 28.751 ± 0.936 months (95% CI: 26.916–30.585) in the low MYH9 group (Fig. 6G and H). Moreover, patients with high expression of both HMGA1 and MYH9 had the shortest survival times, but patients with low expression of both HMGA1 and MYH9 had the longest survival times (Fig. 6I). Further multivariate analysis demonstrated that WHO grade, HMGA1, and MYH9 expression significantly correlated with patient survival (*P* = 0.01, *P* = 0.002, and *P* < 0.001, respectively, Table 2), indicating that these three factors should be treated as independent prognostic factors in patients with glioma.

**Table 2:** Summary of univariate and multivariate Cox regression analysis of overall survival duration.

Factors	Number	Univariate analysis			Multivariate analysis		
		*P*	Hazard ratio	95% confidence interval	*P*	Hazard ratio	95% confidence interval
*Gender*							
Male vs. female	57 vs. 29	0.205	0.147	0.103–0.406			
*Age group*							
<50 vs. ≥50	50 vs. 36	**0.026**	1.977	1.083–3.607			
*WHO grade*							
II vs. III vs. IV	39 vs. 14 vs. 33	**<0.001**	3.382	2.279–5.020	**0.001**	2.212	1.397-3.505
*Pathological diagnosis*							
DA vs. OD vs. AA vs. GBM	28 vs. 11 vs. 14 vs. 33	**<0.001**	2.418	1.777–3.288			
*HMGA1 expression*							
High vs. low	48 vs. 38	**<0.001**	7.568	3.191–17.948	**0.002**	4.290	1.713-10.744
*MYH9 expression*							
High vs. low	49 vs. 37	**<0.001**	7.687	3.369–17.539	**<0.001**	5.572	2.243-13.839

*DA* diffuse astrocytoma, *OD* oligodendroglioma, *AA* Anaplastic astrocytoma, *GBM* glioblastoma.

Bold values indicates statistically significant *P*  <  0.05 values.

## Discussion

The therapeutic challenges presented by gliomas are their high malignancy and infiltrative growth, frequently resulting in incomplete resection and short-term recurrence. These characteristics are closely related to the EMT of tumor cells. The EMT not only promotes the infiltration of tumor cells to the periphery and leads to the residual tumor tissue after surgical resection, but also enables the glioma cells to acquire stem cell-like potentials and chemotherapy resistance [25–27]. Therefore, targeting the EMT in glioma cells has always been an effective strategy.

HMGA1 belongs to the HMG protein family, which alters chromatin structure by direct recognition of the A/T-rich sequences in its target promoter [28]. It is upregulated in gliomas and correlates with patient survival [9]; moreover, knockdown of HMGA1 suppresses the stemness of glioma stem cells and increases their sensitivity to temozolomide (TMZ) [29, 30]. In accordance with these studies, we have found that HMGA1 promoted glioma cell proliferation, invasion, and migration, suggesting that it is a key regulator of the EMT process [10]. Moreover, a new oncogenic ZEB2/miR-637/HMGA1 signaling axis targeting vimentin was identified. Based on this, we further confirmed that HMGA1 inhibition sensitizes glioma cells to TMZ. A subsequent study on the mechanism of HMGA1 showed that HMGA1 upregulated the expression of the PI3K/Akt downstream factors c-Jun and MYH9, while upregulating GSK-3β. These results confirm the relationships among HMGA1, c-Jun, MYH9, and GSK-3β, but further studies on their precise mechanisms are necessary.

MYH9 is a hexamer that is comprised of two heavy chains, two 17 kDa essential light chains, and two 20 kDa modulated light chains [31]. It contains an IQ domain and a myosin head-like domain, which is involved in several important functions, including cytokinesis, cell motility, and maintenance of cell shape [32, 33]. Mutations in this gene have been associated with multiple diseases [12, 34, 35], including cancers [13, 16, 36–40]. MYH9 has been reported to be overexpressed in gastric cancer [41], non-small cell lung cancer [14], colon cancer [40], and breast cancer [42], but was downregulated in squamous cell carcinomas [43]. Moreover, MYH9 promoted the invasion of breast cancer cells [16], and the EGF-dependent phosphorylation of MYH9 resulted in increased migration [44]. However, our understanding of the role of MYH9 in gliomas is limited. It has been reported that MYH9 is deregulated in glioblastoma (GBM) serum small extracellular vesicles and is associated with GBM progression and metastasis [45]. But another study reported that overexpression of MYH9 abrogated the migration-inhibiting effects of the endogenous aryl hydrocarbon receptor agonist, indicating that MYH9 is essential for glioma cell migration [19]. Therefore, the actual function of MYH9 in gliomas is worth exploring. In this study, we found that MYH9 was overexpressed in glioma samples, which promoted glioma cell proliferation, invasion, migration, and TMZ resistance. We further found that HMGA1 increased MYH9 expression through c-Jun-mediated transcription, and MYH9 exerted an oncogenic effect by interacting with GSK-3β, followed by its ubiquitination and degradation. These results indicate that MYH9 plays an essential role in gliomas and as such, presents a potential new therapeutic target.

GSK-3β, which belongs to the serine-threonine kinase family, is involved in embryonic development, cell growth, cell differentiation, apoptosis, and tumorigenesis [46]. In gliomas, GSK-3β is downregulated; its overexpression inhibits angiogenesis and tumor growth by upregulating the β-catenin and mTOR/p70S6K1 pathways [47], and also restores cell differentiation [48]. Moreover, knockdown of GSK-3β induced c-Myc-dependent cell death [49] and promoted glioma stem cell proliferation and colony formation [50]. More interestingly, inhibition of GSK-3β promoted the nuclear translocation of the non-phosphorylated β-catenin, subsequently binding the transcription factors of the TCF/LEF family and activating downstream targets such as MYC, cyclin D1, and BIRC5 [24]. In this study, we found that HMGA1-mediated expression of MYH9 inhibited the expression of GSK-3β and induced the nuclear translocation of non-phosphorylated β-catenin. Therefore, we propose that HMGA1/MYH9 signaling promotes the nuclear translocation of β-catenin via ubiquitinated degradation of GSK-3β.

Our study demonstrated that both HMGA1 and MYH9 promote glioma cell proliferation, invasion, migration, and TMZ resistance. HMGA1 regulated PI3K/Akt/c-Jun signaling, and c-Jun promoted the expression of MYH9. Moreover, MYH9 binds to GSK-3β and promotes its ubiquitinated degradation, resulting in the promotion of malignant phenotypes and chemotherapy-resistant of glioma cells. Therefore, HMGA1 and MYH9 play essential roles in gliomas, which should be considered as tumoral enhancers with significant value as unfavorable progression indicators for patients with glioma, and may serve as feasible therapeutic targets in the future.

## Materials and methods

### Patients and tissue samples

A total of 86 paraffin-embedded glioma specimens and 15 paraffin-embedded adjacent non-tumor specimens were collected from the Department of Neurosurgery at Nanfang Hospital. Tissues from patients who had received preoperative radiation, chemotherapy, or biotherapy were excluded. All patients in this study underwent maximum safe total resection (partly resected cases were excluded). The subjects were 57 males and 29 females, with ages ranging from 24 to 69 (mean, 48.54) years. Prior consent from patients and approval from the Ethics Committees of Nanfang Hospital for Nationalities were obtained for the use of these clinical materials for research purposes. All specimens had a confirmed pathological diagnosis and were classified according to the 2016 World Health Organization (WHO) criteria. The end date of the follow-up study was December 2020, and the median follow-up for overall survival was 19.64 (range, 9–32) months.

### IHC and evaluation of staining

Paraffin sections (4 μm) were deparaffinized and dehydrated, then antigen retrieval was performed in citrate buffer for 3 minutes. Endogenous peroxidase activity and nonspecific antigens were blocked with 3% H_2_O_2_ and goat serum, followed by incubation with HMGA1 antibody (1:200, Abcam) or MYH9 antibody (1:250, Abcam) overnight at 4 °C. After washing, sections were incubated with HRP-conjugated secondary antibodies and visualized using a DAB substrate (Maixin Biotech. Co., Ltd, Fuzhou, China). Immunostaining results were evaluated and scored by two independent pathologists blinded to the clinical parameters, as described in our previous work [10]. Positive nuclear staining scores were defined as: 0, <20%; 1, 20–49%; 2, 50–79%; and 3, >80%. Sums of the cytoplasmic and nuclear staining scores were used as final staining scores (0–12). For statistical analysis, a final staining score of 0–4 and 5–6 in the cytoplasm or 0–3 and 4–6 in the nucleus were considered to be below or high expression, respectively.

### Cell background and transfection

U87 and LN229 were purchased from the Chinese Academy of Sciences (Shanghai, China). U87 and LN229 cells are both MGMT-promoter methylated [51], which is currently the only known biomarker for TMZ response in GBM patients, but these two GBM cell lines are highly resistant to TMZ treatment, indicating another factor rather than MGMT conferred the therapeutic resistance in U87 and LN299 cells. Moreover, the expression of HMGA1 and MYH9 were both high in U87 and LN229 cell lines as shown in Fig. 1J and Fig. 3I. Plasmids and siRNAs were designed and synthesized by RiboBio Co., Ltd. (Guangzhou, China). Lentiviral vectors were designed and synthesized by GeneChem Co., Ltd. (Shanghai, China). Plasmids and siRNAs were transfected into cells using Lipofectamine^TM^ 2000 (Invitrogen Biotechnology Co., Ltd., Shanghai, China) according to the manufacturer’s protocol. Lentiviral vectors were transfected into cells, and polyclonal cells with GFP and puromycin acetyltransferase were selected for further experiments using puromycin. Cells were collected 48-72 hours after transfection for experiments. Interference and overexpression efficiencies were quantified using quantitative real-time PCR (qPCR) and western blotting.

### RT-PCR and qPCR

Total RNA was isolated from cells or fresh clinical tissues using Trizol (TaKaRa Bio, Inc., Shiga, Japan), and cDNA synthesis was performed using reverse transcription reagents (TaKaRa Bio, Inc., Shiga, Japan). Then cDNA was used as a template for amplification with specific primers (Supplemental Table 1). The specificity of the amplification products was confirmed by melting curve analysis. PCR for each gene was repeated thrice. Independent experiments were performed in triplicates.

### Western blotting

Cell-lysate protein concentrations were determined using a BCA protein assay kit (Thermo Scientific, Waltham, MA, USA). Proteins were separated by SDS-PAGE and transferred onto polyvinylidene fluoride membranes, which were probed with primary antibodies: mouse polyclonal Akt, GSK-3β, CDK4, and β-catenin (1:1000; Bioss, Beijing, China); rabbit polyclonal p-Akt, vimentin, cyclin D1, MYH9, c-Jun, vimentin, Sox2, and Oct-4 (1:1000; Bioss, Beijing, China), and rabbit polyclonal PI3K, p-PI3K, and HMGA1 (1:1000; Cell Signaling Technology, Danvers, USA). Rabbit monoclonal anti-β-actin antibody (1:1000; CoWin Bioscience, Beijing, China) was used for normalization. HRP-conjugated anti-rabbit or anti-mouse IgG antibodies were used as secondary antibodies (1:2000; CoWin Bioscience, Beijing, China). Proteins were detected using an enhanced chemiluminescence reagent (Thermo Scientific, Waltham, MA, USA). Images were captured using a ChemiDoc^TM^ CRS + Molecular Imager (Bio-Rad, Hercules, CA, USA). Grayscale semi-quantification of the bands is shown in Supplemental Fig. 4 for all western blots.

### Immunofluorescence and confocal microscopy

Cells were plated on coverslips in 48-well plates and cultured overnight to allow them to adhere. After fixation with 4% paraformaldehyde and permeabilization with 0.2% Triton X-100, cells were incubated with mouse polyclonal anti-GSK-3β and rabbit polyclonal anti-MYH9 antibodies (1:100; Bioss, Beijing, China). Cells were counterstained with 0.2 mg/ml 4′,6-diamidino-2-phenylindole (DAPI) and visualized using a fluorescence confocal microscope (Carl Zeiss LSM800).

### Migration and invasion assay

A Transwell assay (BD Biosciences, NJ, USA) was performed to detect cell migration and invasion abilities. Cells were suspended in 100 μl Dulbecco’s Modified Eagle Medium (DMEM) without serum and seeded into the top chamber of the Transwell coated with Matrigel (BD Biosciences, NJ, USA) or left uncoated, and the bottom chambers were filled with 500 μl DMEM supplemented with 10% fetal bovine serum. The migrated cells were stained with crystal violet, photographed, and quantified by counting the cell number in five random fields.

### MTT and EdU incorporation assay

Cell proliferation and drug sensitivity were tested using the MTT assay. Exponentially growing cells were seeded into 96-well plates and cultured overnight to allow for cell adherence. Cell viability was measured using MTT (5 mg/ml) (Sigma-Aldrich, MO, USA). EdU incorporation was performed using an Apollo567 in vitro imaging kit (RiboBio Co., Ltd, Guangzhou, China) according to the manufacturer’s protocol. Cells were incubated with 10 μM EdU for 2 h, then fixed with 4% paraformaldehyde. After permeabilization with 0.3% Triton X-100, cells were stained with Apollo fluorescent dyes and 5 μg/ml DAPI.

### TMZ chemosensitivity

Transfected U87 and LN229 cells were seeded at a density of 6000 cells per well, and after 24 h the cells were treated with different concentrations (50, 100, 200, and 400 μM) of TMZ (Merck product) for 48 h. MTT assays were then performed to determine the fraction of cells that survived TMZ exposure. The resistance of the induced cells was measured using the IC50. The TMZ concentration causing 50% inhibition of glioma cell activity was defined as the IC50.

### In vivo orthotopic xenograft study

Animal protocols were approved by the Institutional Animal Ethics Committee of the Experimental Animal Center of Nanfang Hospital. Female nude mice (4 weeks old) were purchased from Southern Medical University and divided into two groups of five mice per group. After acclimation for 1 week, the mice were anesthetized with 1% pentobarbital sodium and placed in a stereotactic fixation device (RWD Life Science, Shenzhen, China) prior to xenograft inoculation. A total of 10^6^ cells in 5 µl of sterile PBS were stereotactically implanted into the point located 1 mm posterior to the bregma, 2 mm left of the midline, and 2.5 mm deep into the brain. Cells were automatically injected by a stereotactic fixation device over 5 minutes. The mice were sacrificed when the tumors of control groups were grown into the appropriate size confirming by animal MRI. The mice brains were then fixed in 4% paraformaldehyde for 24 hours and embedded in paraffin for further staining.

### Dual-luciferase reporter assay

A fragment of the MYH9 promoter was amplified by PCR. Mutagenesis of the c-Jun binding site was performed using the GeneTailor Site-Directed Mutagenesis System (Invitrogen, Guangzhou, China). The wild-type and mutant promoters were separately cloned into the psiCHECK-2 vector. The construct was cotransfected with si-c-Jun and c-Jun plasmids (or their control plasmids) into cells. Luciferase activity was measured 48 h after transfection using the Dual-Luciferase Reporter Assay System (Promega Corporation, Madison, WI, USA).

### Chromatin immunoprecipitation

ChIP assays were performed using a commercial assay kit (Thermo Scientific, Waltham, MA, USA) according to the manufacturer’s protocol. Chromatin was cross-linked, isolated, and digested with micrococcal nuclease to obtain DNA fragments. Anti-c-Jun (ChIP Grade, Abcam) or IgG was added for immunoprecipitation. After elution and purification, the recovered DNA fragments were subjected to RT-PCR and qPCR. IgG served as a negative control.

### Co-immunoprecipitation assay

Co-IP was carried out using a Pierce Co-Immunoprecipitation kit (Thermo Scientific, Waltham, MA, USA) according to the manufacturer’s instructions. Briefly, total protein was extracted from cells and quantified. A total of 5 mg of protein was incubated with 10 μg specific antibodies (Abcam) or IgG overnight at 4 °C. After elution, the recovered proteins were subjected to silver staining, mass spectrometry, and western blotting. IgG was used as a negative control.

### Statistical analysis

All experiments were replicated at least three times. All data were analyzed using SPSS (version 26.0; SPSS Inc. Chicago, IL, USA) and GraphPad Prism v9.0 (GraphPad Software, San Diego, CA, USA) software. Data are expressed as means ± SD from at least three independent experiments. Statistical significance was determined using the Student’s two-tailed *t* test for two groups, one-way ANOVA for multiple groups, and a general linear model for tumor growth and MTT assays. Correlations between gene expression and clinicopathological characteristics were analyzed using the chi-square test. Log-rank tests were performed on Kaplan–Meier survival curves to elucidate relationships between gene expression and overall survival in patients. Univariate and multivariate survival analyses were performed using the Cox proportional hazards regression model. All statistical tests were two-sided. A *P* value of <0.05 was considered to be statistically significant.

## Supplementary information

Supplemental Tables

Supplemental Figure legends

Supplemental Figure 1

Supplemental Figure 2

Supplemental Figure 3

Supplemental Figure 4

## Data Availability

The data sets used and/or analyzed during the current study are available from the corresponding author on reasonable request.

## References

[1] Maher EA (2001). Malignant glioma: genetics and biology of a grave matter. Genes Dev.

[2] Stupp R, Hegi ME, Mason WP, van den Bent MJ, Taphoorn MJ, Janzer RC (2009). Effects of radiotherapy with concomitant and adjuvant temozolomide versus radiotherapy alone on survival in glioblastoma in a randomised phase III study: 5-year analysis of the EORTC-NCIC trial. Lancet Oncol.

[3] Emdad L, Dent P, Sarkar D, Fisher PB (2012). Future approaches for the therapy of malignant glioma: targeting genes mediating invasion. Future Oncol.

[4] Lefranc F, Brotchi J, Kiss R (2005). Possible future issues in the treatment of glioblastomas: special emphasis on cell migration and the resistance of migrating glioblastoma cells to apoptosis. J Clin Oncol.

[5] Zhao C, Li Y, Zhang W, Zhao D, Ma L, Ma P (2018). IL17 induces NSCLC A549 cell proliferation via the upregulation of HMGA1, resulting in an increased cyclin D1 expression. Int J Oncol.

[6] Zhang S, Lei R, Wu J, Shan J, Hu Z, Chen L (2017). Role of high mobility group A1 and body mass index in the prognosis of patients with breast cancer. Oncol Lett.

[7] Fu F, Wang T, Wu Z, Feng Y, Wang W, Zhou S (2018). HMGA1 exacerbates tumor growth through regulating the cell cycle and accelerates migration/invasion via targeting miR-221/222 in cervical cancer. Cell Death Dis.

[8] Wang J, Xu X, Mo S, Tian Y, Wu J, Zhang J (2016). Involvement of microRNA-1297, a new regulator of HMGA1, in the regulation of glioma cell growth in vivo and in vitro. Am J Transl Res.

[9] Pang B, Fan H, Zhang IY, Liu B, Feng B, Meng L (2012). HMGA1 expression in human gliomas and its correlation with tumor proliferation, invasion and angiogenesis. J Neurooncol.

[10] Zeng Y, Que T, Lin J, Zhan Z, Xu A, Wu Z (2021). Oncogenic ZEB2/miR-637/HMGA1 signaling axis targeting vimentin promotes the malignant phenotype of glioma. Mol Ther Nucleic Acids.

[11] Vicente-Manzanares M, Ma X, Adelstein RS, Horwitz AR (2009). Non-muscle myosin II takes centre stage in cell adhesion and migration. Nat Rev Mol Cell Biol.

[12] Pecci A, Ma X, Savoia A, Adelstein RS (2018). MYH9: structure, functions and role of non-muscle myosin IIA in human disease. Gene.

[13] Liang S, He L, Zhao X, Miao Y, Gu Y, Guo C (2011). MicroRNA let-7f inhibits tumor invasion and metastasis by targeting MYH9 in human gastric cancer. PLoS One.

[14] Katono K, Sato Y, Jiang SX, Kobayashi M, Nagashio R, Ryuge S (2015). Prognostic significance of MYH9 expression in resected non-small cell lung cancer. PLoS One.

[15] Mu Y, Chen Y, Zhang G, Zhan X, Li Y, Liu T (2013). Identification of stromal differentially expressed proteins in the colon carcinoma by quantitative proteomics: proteomics and 2DE. Electrophoresis.

[16] Derycke L, Stove C, Vercoutter-Edouart AS, De Wever O, Dolle L, Colpaert N (2011). The role of non-muscle myosin IIA in aggregation and invasion of human MCF-7 breast cancer cells. Int J Dev Biol.

[17] Ye G, Yang Q, Lei X, Zhu X, Li F, He J (2020). Nuclear MYH9-induced CTNNB1 transcription, targeted by staurosporin, promotes gastric cancer cell anoikis resistance and metastasis. Theranostics.

[18] Lin X, Li A, Li Y-H, Luo R-C, Zou Y-J, Liu Y-Y (2020). Silencing MYH9 blocks HBx-induced GSK3β ubiquitination and degradation to inhibit tumor stemness in hepatocellular carcinoma. Sig Transduct Target Ther.

[19] Zhao L, Shu Q, Sun H, Ma Y, Kang D, Zhao Y (2020). 1’H-indole-3’-carbonyl-thiazole-4-carboxylic acid methyl ester blocked human glioma cell invasion via aryl hydrocarbon receptor’s regulation of cytoskeletal contraction. Biomed Res Int.

[20] Beadle C, Assanah MC, Monzo P, Vallee R, Rosenfeld SS, Canoll P (2008). The role of myosin II in glioma invasion of the brain. Mol Biol Cell.

[21] Wang Y, Qian T, You G, Peng X, Chen C, You Y (2015). Localizing seizure-susceptible brain regions associated with low-grade gliomas using voxel-based lesion-symptom mapping. Neuro-Oncol.

[22] Liu X, Li Y, Qian Z, Sun Z, Xu K, Wang K (2018). A radiomic signature as a non-invasive predictor of progression-free survival in patients with lower-grade gliomas. NeuroImage: Clin.

[23] Yellaboina S, Tasneem A, Zaykin DV, Raghavachari B, Jothi R (2011). DOMINE: a comprehensive collection of known and predicted domain-domain interactions. Nucleic Acids Res.

[24] Sareddy GR, Panigrahi M, Challa S, Mahadevan A, Babu PP (2009). Activation of Wnt/beta-catenin/Tcf signaling pathway in human astrocytomas. Neurochem Int.

[25] Que T, Song Y, Liu Z, Zheng S, Long H, Li Z (2015). Decreased miRNA-637 is an unfavorable prognosis marker and promotes glioma cell growth, migration and invasion via direct targeting Akt1. Oncogene.

[26] Iser IC, Pereira MB, Lenz G, Wink MR (2017). The epithelial-to-mesenchymal transition-like process in glioblastoma: an updated systematic review and in silico investigation. Med Res Rev.

[27] Tao C, Huang K, Shi J, Hu Q, Li K, Zhu X (2020). Genomics and prognosis analysis of epithelial-mesenchymal transition in glioma. Front Oncol.

[28] Reeves R (2000). Structure and function of the HMGI(Y) family of architectural transcription factors. Environ Health Perspect.

[29] Colamaio M, Tosti N, Puca F, Mari A, Gattordo R, Kuzay Y (2016). HMGA1 silencing reduces stemness and temozolomide resistance in glioblastoma stem cells. Expert Opin Ther Targets.

[30] Lopez-Bertoni H, Lal B, Michelson N, Guerrero-Cazares H, Quinones-Hinojosa A, Li Y (2016). Epigenetic modulation of a miR-296-5p:HMGA1 axis regulates Sox2 expression and glioblastoma stem cells. Oncogene.

[31] Sellers JR (2000). Myosins: a diverse superfamily. Biochim Biophys Acta.

[32] Golomb E, Ma X, Jana SS, Preston YA, Kawamoto S, Shoham NG (2004). Identification and characterization of nonmuscle myosin II-C, a new member of the myosin II family. J Biol Chem.

[33] Krendel M, Mooseker MS (2005). Myosins: tails (and heads) of functional diversity. Physiol (Bethesda).

[34] Newell-Litwa KA, Horwitz R, Lamers ML (2015). Non-muscle myosin II in disease: mechanisms and therapeutic opportunities. Dis Model Mech.

[35] Marini M, Bruschi M, Pecci A, Romagnoli R, Musante L, Candiano G (2006). Non-muscle myosin heavy chain IIA and IIB interact and co-localize in living cells: relevance for MYH9-related disease. Int J Mol Med.

[36] Chiu HC, Chang TY, Huang CT, Chao YS, Hsu JT (2012). EGFR and myosin II inhibitors cooperate to suppress EGFR-T790M-mutant NSCLC cells. Mol Oncol.

[37] Xia ZK, Yuan YC, Yin N, Yin BL, Tan ZP, Hu YR (2012). Nonmuscle myosin IIA is associated with poor prognosis of esophageal squamous cancer. Dis Esophagus.

[38] Xu Z, Li P, Wei D, Wang Z, Bao Y, Sun J (2016). NMMHC-IIA-dependent nuclear location of CXCR4 promotes migration and invasion in renal cell carcinoma. Oncol Rep.

[39] Yu M, Wang J, Zhu Z, Hu C, Ma Q, Li X (2016). Prognostic impact of MYH9 expression on patients with acute myeloid leukemia. Oncotarget.

[40] Liao Q, Li R, Zhou R, Pan Z, Xu L, Ding Y (2017). LIM kinase 1 interacts with myosin-9 and alpha-actinin-4 and promotes colorectal cancer progression. Br J Cancer.

[41] Ye G, Huang K, Yu J, Zhao L, Zhu X, Yang Q (2017). MicroRNA-647 targets SRF-MYH9 axis to suppress invasion and metastasis of gastric cancer. Theranostics.

[42] Betapudi V, Licate LS, Egelhoff TT (2006). Distinct roles of nonmuscle myosin II isoforms in the regulation of MDA-MB-231 breast cancer cell spreading and migration. Cancer Res.

[43] Schramek D, Sendoel A, Segal JP, Beronja S, Heller E, Oristian D (2014). Direct in vivo RNAi screen unveils myosin IIa as a tumor suppressor of squamous cell carcinomas. Science.

[44] Dulyaninova NG, House RP, Betapudi V, Bresnick AR (2007). Myosin-IIA heavy-chain phosphorylation regulates the motility of MDA-MB-231 carcinoma cells. Mol Biol Cell.

[45] Anastasi F, Greco F, Dilillo M, Vannini E, Cappello V, Baroncelli L (2020). Proteomics analysis of serum small extracellular vesicles for the longitudinal study of a glioblastoma multiforme mouse model. Sci Rep.

[46] Grimes CA, Jope RS (2001). The multifaceted roles of glycogen synthase kinase 3beta in cellular signaling. Prog Neurobiol.

[47] Zhao P, Li Q, Shi Z, Li C, Wang L, Liu X (2015). GSK-3β regulates tumor growth and angiogenesis in human glioma cells. Oncotarget.

[48] Li Y, Lu H, Huang Y, Xiao R, Cai X, He S (2010). Glycogen synthase kinases-3beta controls differentiation of malignant glioma cells. Int J Cancer.

[49] Kotliarova S, Pastorino S, Kovell LC, Kotliarov Y, Song H, Zhang W (2008). Glycogen synthase kinase-3 inhibition induces glioma cell death through c-MYC, nuclear factor-kappaB, and glucose regulation. Cancer Res.

[50] Gürsel DB, Banu MA, Berry N, Marongiu R, Burkhardt J-K, Kobylarz K (2015). Tight regulation between cell survival and programmed cell death in GBM stem-like cells by EGFR/GSK3b/PP2A signaling. J Neurooncol.

[51] Lee SY (2016). Temozolomide resistance in glioblastoma multiforme. Genes Dis.

