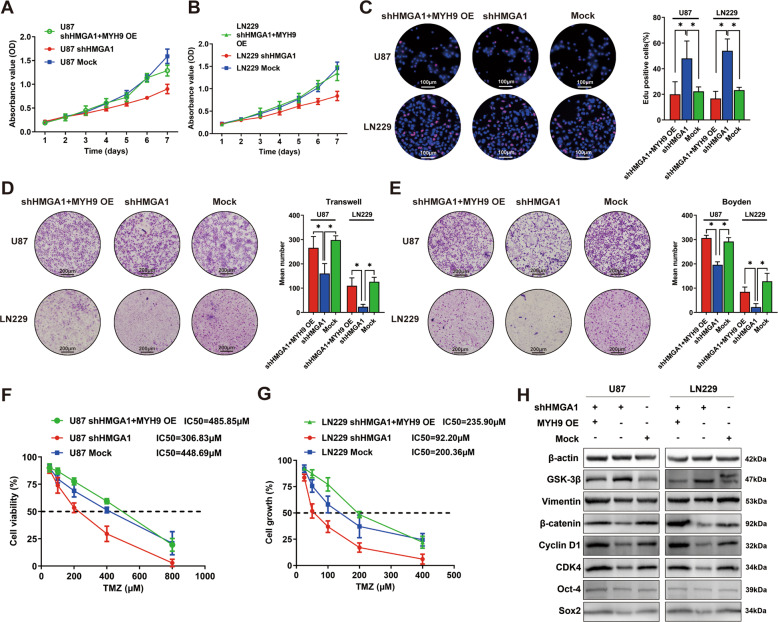# Correction to: HMGA1 stimulates MYH9-dependent ubiquitination of GSK-3β via PI3K/Akt/c-Jun signaling to promote malignant progression and chemoresistance in gliomas

**DOI:** 10.1038/s41419-022-04547-9

**Published:** 2022-02-21

**Authors:** Tianshi Que, Haojie Zheng, Yu Zeng, Xinru Liu, Ge Qi, Qingcuo La, Tuo Liang, Zhiyong Li, Guozhong Yi, Shichao Zhang, Junjie Li, Jing Nie, Jian-er Tan, Guanglong Huang

**Affiliations:** 1 Department of Neurosurgery, Nanfang Hospital, Southern Medical University, Guangzhou, 510515 Guangdong People’s Republic of China; 2 The Laboratory for Precision Neurosurgery, Nanfang Hospital, Southern Medical University, Guangzhou, 510515 Guangdong People’s Republic of China; 3 Nanfang Glioma Center, Nanfang Hospital, Southern Medical University, Guangzhou, 510515 Guangdong People’s Republic of China; 4 Department of Cell Biology, School of Basic Medical Sciences, Southern Medical University, Guangzhou, 510515 Guangdong People’s Republic of China; 5 The First Clinical Medical School, Southern Medical University, Guangzhou, 510515 Guangdong People’s Republic of China; 6 School of Pharmaceutical Sciences, Southern Medical University, Guangzhou, 510515 Guangdong People’s Republic of China; 7 Nanfang PET Center, Nanfang Hospital, Southern Medical University, Guangzhou, 510515 Guangdong People’s Republic of China

**Keywords:** CNS cancer, Cell signalling

Correction to: *Cell Death & Disease* 10.1038/s41419-021-04440-x, published online 10 December 2021

The original version of this article unfortunately contained a mistake. The authors found that two images in Fig. 3F and Fig. 4D were the same. The authors apologize for the error. The original article has been corrected.