# Supplementary material for: HMGA1 stimulates MYH9-dependent ubiquitination of GSK-3β via PI3K/Akt/c-Jun signaling to promote malignant progression and chemoresistance in gliomas
Source: Cell Death Dis. 2021 Dec 10;12(12):1147. doi: 10.1038/s41419-021-04440-x (PMC8660812; doi:10.1038/s41419-021-04440-x)
Supplement: Supplementary file 1 — Supplemental Tables [file 41419_2021_4440_MOESM1_ESM.docx]

Supplemental Table 1. The primer sequences used in this study.

| **Primers** | | **Sequences (5’-3’)** |
| --- | --- | --- |
| HMGA1 | Forward | ATGAACTCCGAAGGCCAGCC |
|  | Reverse | CCTTCCTAGGTCTGCCTCTTGG |
| c-Jun | Forward | CCTGCGTCTTAGGCTTCTCC |
|  | Reverse | GCTCGCCCAAGTTCAACAA |
| MYH9 | Forward | AGTTTGTCTCGGAGCTGTGG |
|  | Reverse | GGTTCGTGTTCCTCAGCGTA |
| GSK-3β | Forward | GTCCGATTGCGTTATTTC |
|  | Reverse | AAGAGGTTCTGCGGTTTA |
| ARF5 | Forward | CTCGCTGTCCACCTTCCA |
|  | Reverse | ACCTTCACCGTTCCAGTTTT |
| GSK-3β 15K | Forward | TTTGCGGAGAGCTGCGATCCGGTGCAGCAGCCTTCAGCTTTTG |
|  | Reverse | AGGCTGCTGCACCGGATCGCAGCTCTCCGCAAAGGAGGTGGTT |
| GSK-3β 27K | Forward | GCTTTTGGCAGCATGGAGGTTAGCAGAGACAAGGACGGCAGCAAGGTGACAA |
|  | Reverse | CTTGTCTCTGCTAACCTCCATGCTGCCAAAAGCTGAAGGCTGCT |
| GSK-3β 36K | Forward | GACAAGGACGGCAGCGGTGTGACAACAGTGGTGGCAACTCCTGG |
|  | Reverse | CACCACTGTTGTCACACCGCTGCCGTCCTTGTCTCTGCTAACTTT |
| MYH9 mut1 | Forward | ATAGCACGAGATTTCTCTCGGCCTGGGCCAGCGGATTGTT GATGAA |
|  | Reverse | ATCCGCTGGCCCAGGCCGAGAGAAATCTCGTGCTATCCGCCAAGCCAA |
| MYH9 mut2 | Forward | CACAGTGCGGAACATGCGAGATAATAGAAGATGTGGAAGGTCCGTTCT |
|  | Reverse | ACATCTTCTATTATCTCGCATGTTCCGCACTGTGGGGCAG |
