# Supplementary material for: HMGA1 stimulates MYH9-dependent ubiquitination of GSK-3β via PI3K/Akt/c-Jun signaling to promote malignant progression and chemoresistance in gliomas
Source: Cell Death Dis. 2021 Dec 10;12(12):1147. doi: 10.1038/s41419-021-04440-x (PMC8660812; doi:10.1038/s41419-021-04440-x)
Supplement: Supplementary file 2 — Supplemental Figure legends [file 41419_2021_4440_MOESM2_ESM.docx]

**Supplemental Figure 1.** Relative results of Western blot assay and chromatin immunoprecipitation (ChIP) assay. **A.** Knockdown of HMGA1 promoted the expression of γ-H2AX. **B.** The level of PI3K/Akt signaling in U87 and LN229 cells after HMGA1 overexpression. **C.** RT-PCR after ChIP assay confirmed that potential protein-DNA interactions between c-Jun and the transcription regulatory region of MYH9 in glioblastoma cells. **D.** Knockdown of MYH9 promoted the expression of γ-H2AX. **E.** MYH9 overexpression could reversed the shHMGA1-mediated reduction of the γ-H2AX.

**Supplemental Figure 2.** Overexpression of MYH9 enhanced proliferation, invasion, migration and TMZ-resistance of glioma cells. **A and B.** MTT assays showed MYH9 upregulation promoted the cell growth. **C.** Edu incorporation assays showed MYH9 promoted the cell proliferation by blocking G1/S phase transition. **D and E.** Transwell (D) and Boyden (E) chamber assays confirmed MYH9 increased the ability of cell invasion and migration. **F and G.** Dose-response curves were delineated for TMZ treatment in MYH9-overexpression cells and the control groups to identify the role of MYH9 in chemoresistance. **H.** Western blot assay confirmed the change of downstream pathway after MYH9 upregulation.

**Supplemental Figure 3.** Upregulated HMGA1 could promote the cytoplasmic expression of MYH9, and synchronously increased the total expression and the nuclear expression of β-catenin.

**Supplemental Figure 4.** Greyscale semi-quantification of all bands for all western blot experiments. **A.** Figure 1J. **B.** Figure 2D. **C.** Figure 3I. **D.** Figure 4H. **E.** Figure 5B. **F.** Figure 5C. **G.** Figure 5D. **H.** Figure 5E. **I.** Supplemental Figure 1A. **J.** Supplemental Figure 1B. **K.** Supplemental Figure 1D. **L.** Supplemental Figure 1E. **M.** Supplemental Figure 2H.
