# Supplementary figures and images for: HMGA1 stimulates MYH9-dependent ubiquitination of GSK-3β via PI3K/Akt/c-Jun signaling to promote malignant progression and chemoresistance in gliomas
Source: Cell Death Dis. 2021 Dec 10;12(12):1147. doi: 10.1038/s41419-021-04440-x (PMC8660812; doi:10.1038/s41419-021-04440-x)

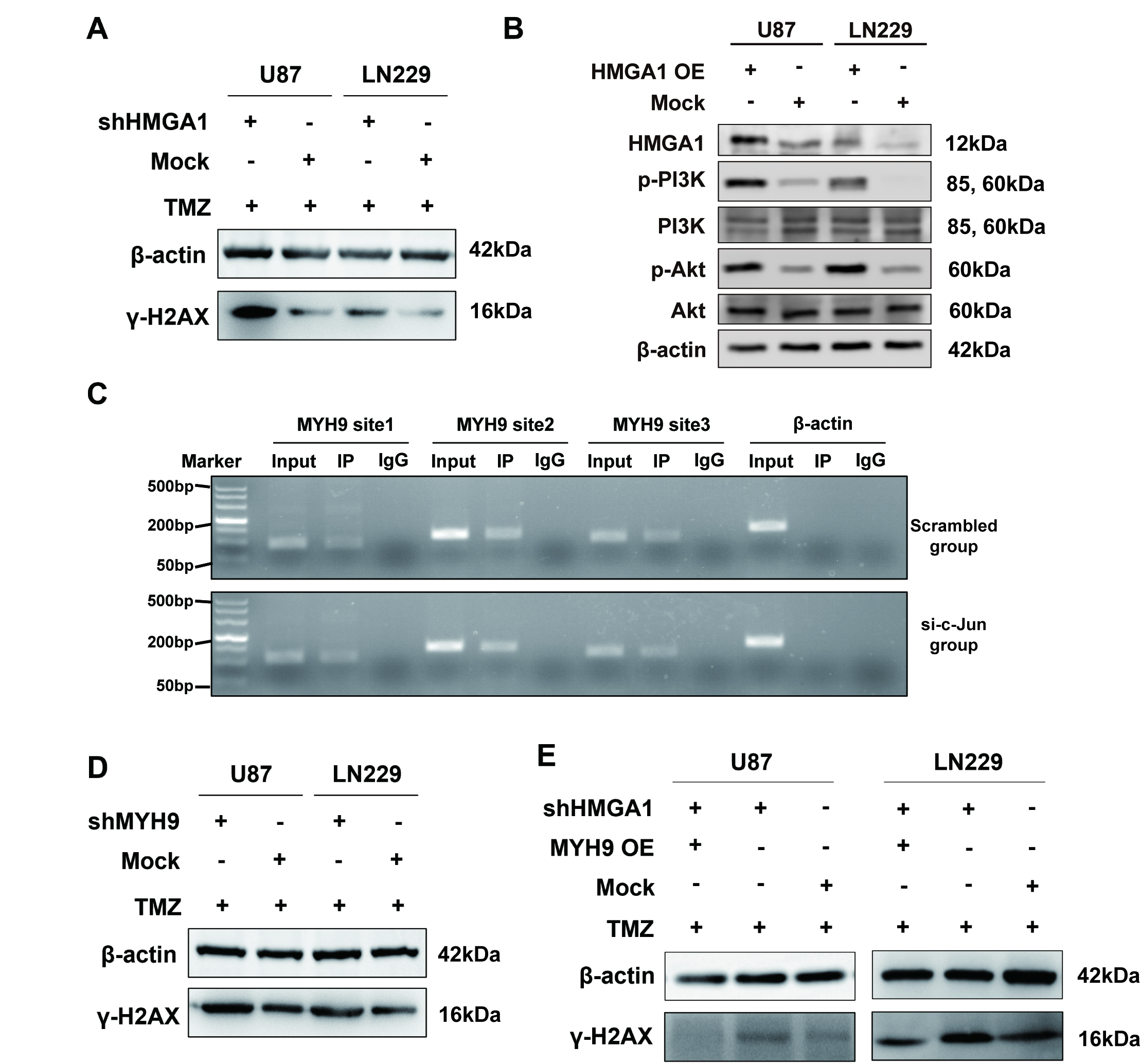

Supplement: Supplementary file 3 — Supplemental Figure 1 [file 41419_2021_4440_MOESM3_ESM.tif]

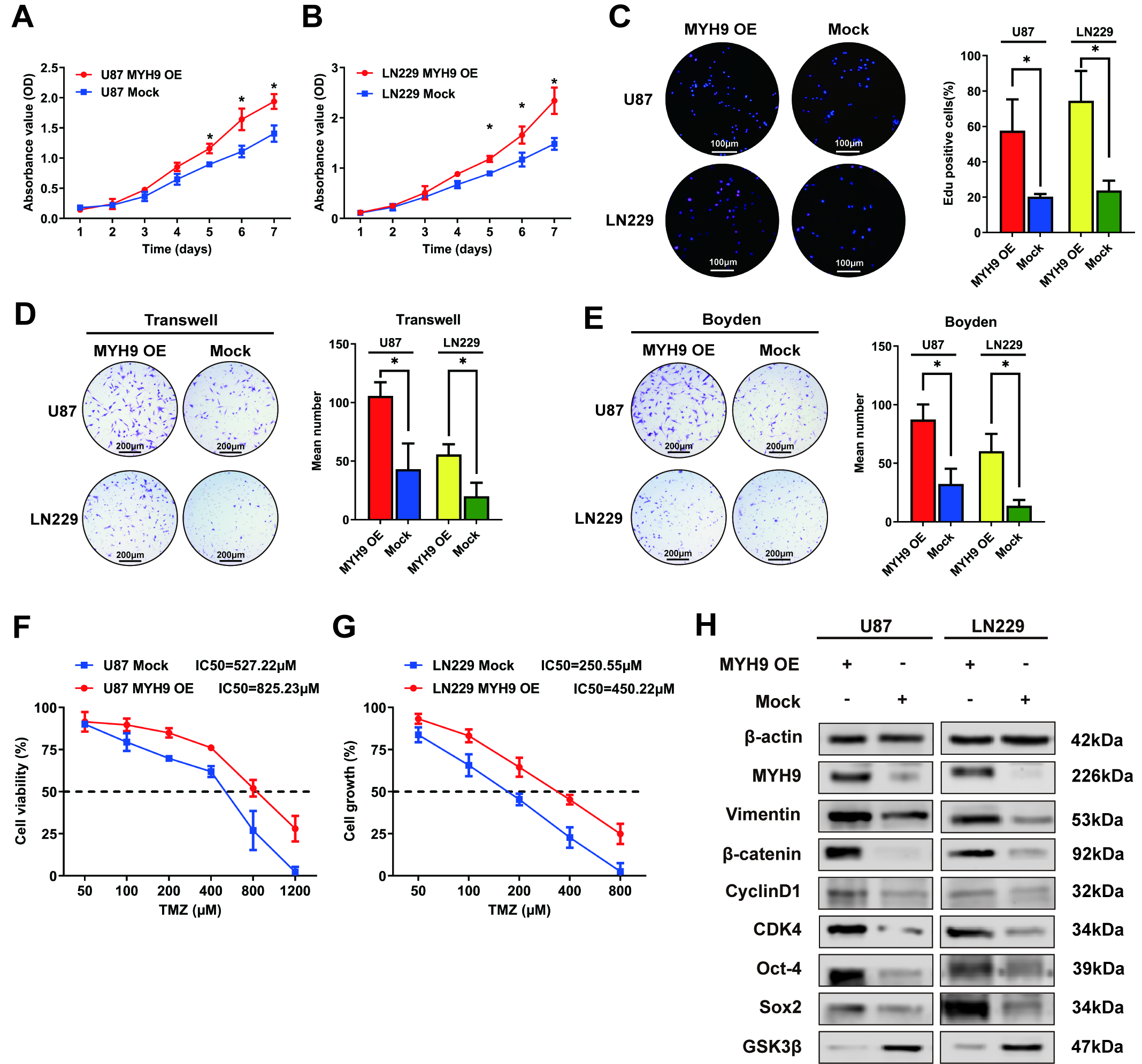

Supplement: Supplementary file 4 — Supplemental Figure 2 [file 41419_2021_4440_MOESM4_ESM.tif]

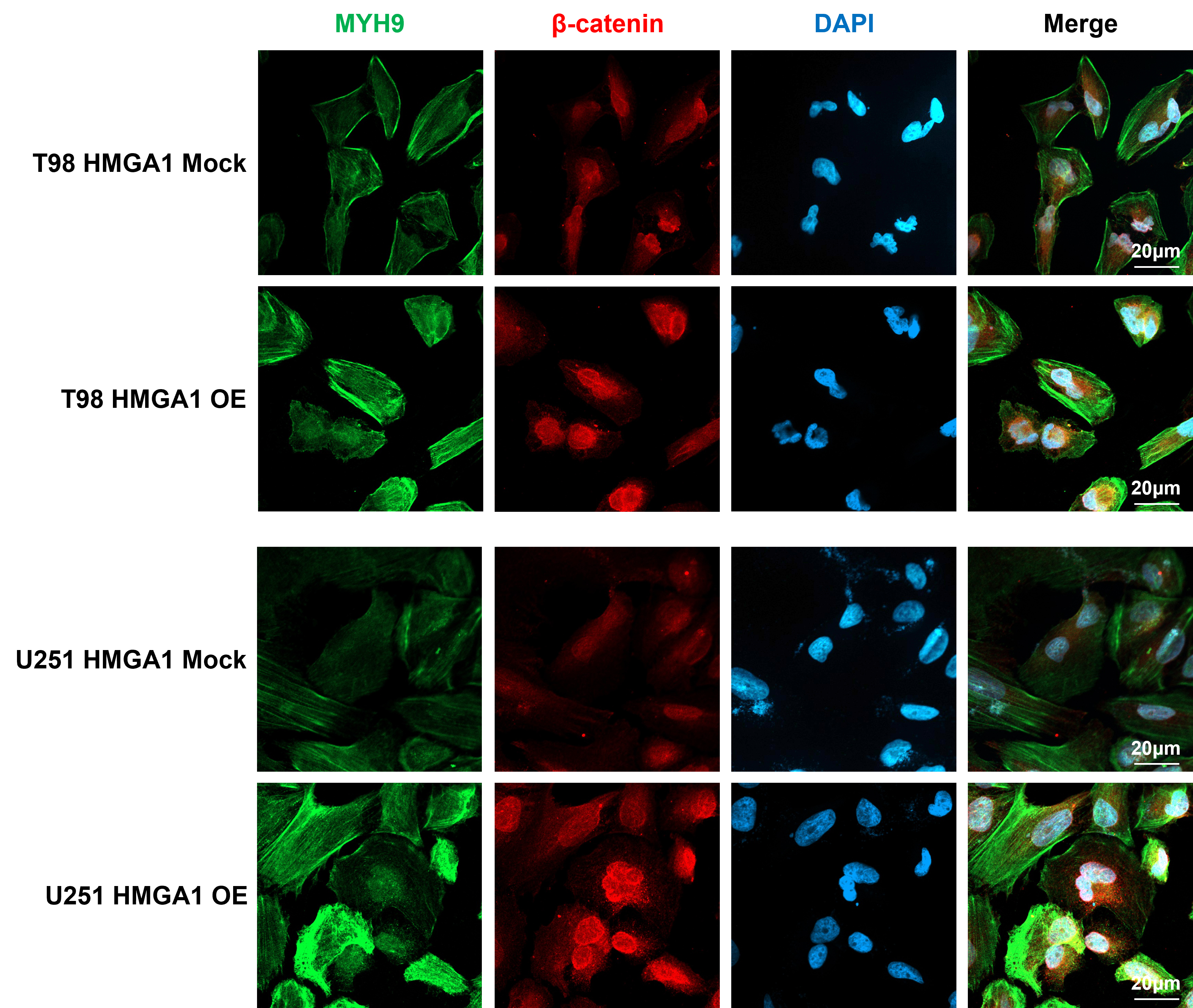

Supplement: Supplementary file 5 — Supplemental Figure 3 [file 41419_2021_4440_MOESM5_ESM.tif]

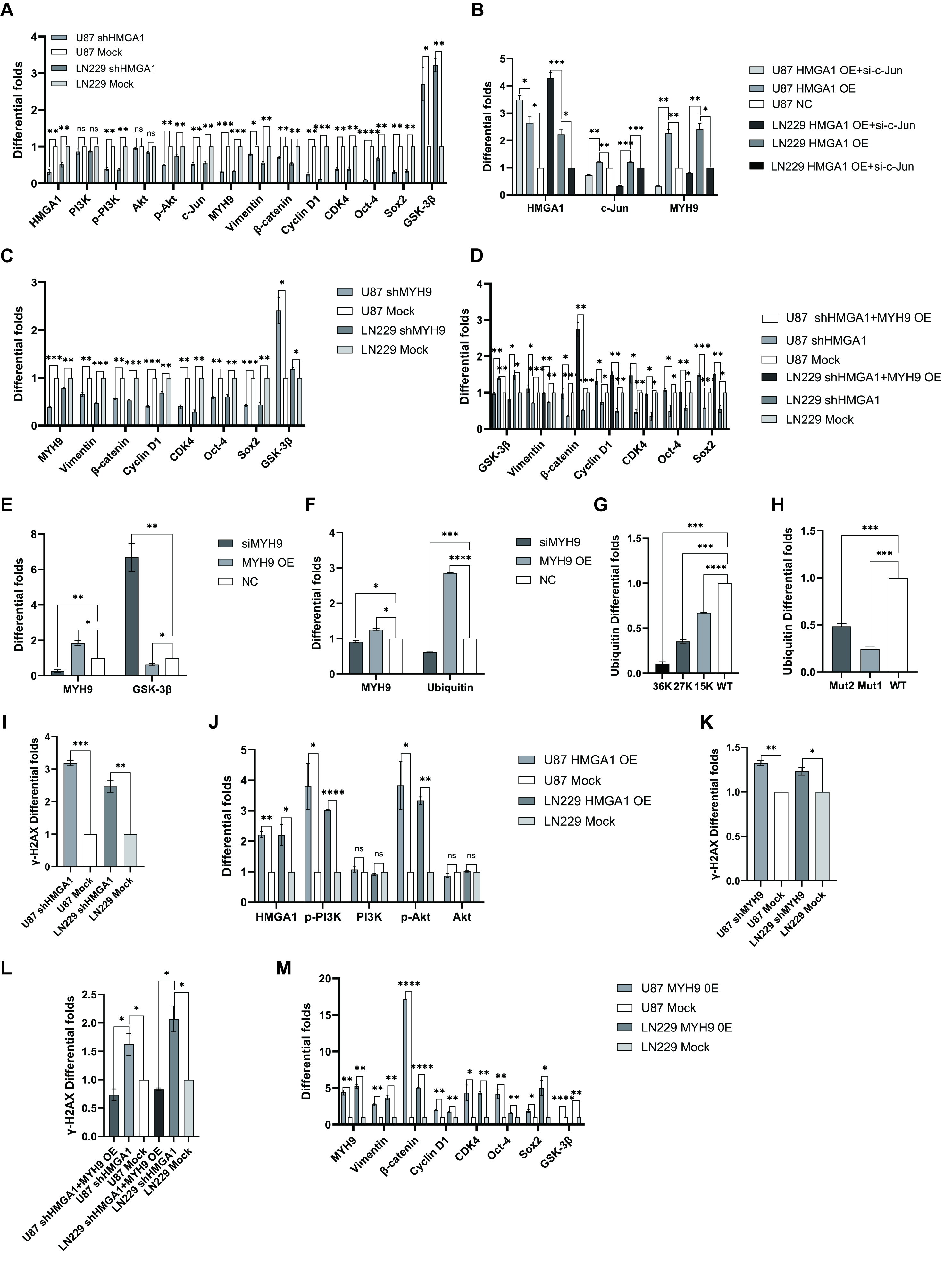

Supplement: Supplementary file 6 — Supplemental Figure 4 [file 41419_2021_4440_MOESM6_ESM.tif]
